# Changing the way we care: restoring Mana to Māori mothers through kaupapa Māori antenatal practice

**DOI:** 10.3389/fgwh.2026.1781546

**Published:** 2026-06-03

**Authors:** Dianne Te Tau

**Affiliations:** Counties Manukau Health, Manukau City/Auckland, New Zealand

**Keywords:** Kaupapa Māori, māori maternal health, antenatal care, cultural safety, whānau-centred care, HEAL model

## Abstract

Abstract Māori women in Aotearoa New Zealand continue to encounter maternity and early parenting systems shaped by surveillance-based practice, deﬁcit assumptions, and the enduring impacts of colonisation. These structures contribute to mistrust, delayed antenatal engagement, and disproportionate child protection intervention. This study explores wāhine Māori experiences of Te Waiora, a Māori-led antenatal support model grounded in the HEAL framework (Honesty, Empowerment, Aspirations, Learning) and informed by Māori health models including Te Whare Tapa Whā and Te Wheke. Using a Kaupapa Māori qualitative research design, semi-structured interviews were conducted with participants involved in Te Waiora in the Counties Manukau region. Fourteen participants contributed to the study: seven wāhine Māori, two midwives, two community health workers, one senior social work advocate, and two legal advocates. One wāhine Māori participant exited the study prior to completing data collection due to whānau matters. Data were analysed using a thematic approach informed by kaupapa Māori interpretive principles. Participants described increased trust in providers, strengthened antenatal engagement, reduced fear of statutory intervention, and sustained connection with their pēpi and whānau. These ﬁndings represent participant-reported experiences and perceived impacts rather than causal evidence of model effectiveness. The study contributes qualitative insight into how Indigenous-led, whānau-centred antenatal care is experienced as safer and more supportive than surveillance-based research maternity approaches and adds to the growing body of Indigenous maternal health scholarship. Deﬁnitions are informed by Māori health frameworks (Durie, 2001; Pere, 1991; Smith, 1999) and Te Waiora HEAL practice (Te Waiora, 2018).

## Introduction

Māori continue to have higher birth rates than non-Māori in Aotearoa New Zealand, with wāhine Māori experiencing the youngest median age of childbirth and the highest rate of births per 1,000 women of reproductive age (1). Despite long-standing whānau-centred caregiving traditions and intergenerational knowledge of pregnancy and child-rearing, Māori mothers are signiﬁcantly more likely to enter maternity systems under heightened scrutiny. They are disproportionately categorised as “high-risk,” subjected to intensiﬁed monitoring, and exposed to child protection assessment during pregnancy or in the immediate post-natal period (17; Table 1).

**Table 1:** Glossary of Māori terms used within the context of this study.

Māori Term	Deﬁnition (Contextual to this Study)
Aroha	Compassionate, relational care grounded in empathy, respect, and connection. Not sentiment, but an active practice of care.
Hapū	Pregnant; also refers to sub-tribe. Used here primarily to describe pregnancy.
Hinengaro	The mind, emotions, and inner psychological world.
Kaimahi	Workers or practitioners, particularly those working in community, health, or social support roles.
Karakia	Prayer or spiritual incantation used to open or close space and ensure collective grounding.
Kaupapa Māori	Māori-led approaches grounded in Māori philosophies, values, and ways of knowing and being.
Mana	Inherent authority, dignity, power, and spiritual presence. Mana is intrinsic and not granted by the state or services.
Mana Ake	The uniqueness and strength inherent in each person. Recognises the distinctive identity, gifts, and potential of each child and adult.
Manaakitanga	The practice of care, hospitality, and upholding the dignity of others through relational responsibility.
Māmā	Mother/mother ﬁgure.
Mauri	Life force or vitality present within all living beings.
Pēpi	Baby/infant.
Rangatahi	Young parents or young people.
Taha	A dimension or pillar of wellbeing (used in Te Whare Tapa Whā).
Taha Tinana	Physical health and bodily wellbeing.
Taha Hinengaro	Emotional and mental wellbeing.
Taha Whānau	Whānau relationships, support, identity, and belonging.
Taha Wairua	Spiritual wellbeing and sense of connection to self, ancestors, and place.
Tapu	Sacredness, dignity, or restriction that protects the wellbeing of a person or place.
Te Ao Māori	The Māori worldview, encompassing cultural, social, spiritual, and relational understandings of life.
Te Ao Mārama	A state of clarity, understanding, or enlightenment; used in this study to describe movement toward strength and conﬁdence.
Te Kore	A state of uncertainty, potential, or absence of clarity Represents

National data demonstrate that Māori infants are over-represented in care and protection notiﬁcations and newborn uplifts, particularly within the ﬁrst days and weeks of life (2, 18).

These patterns are not indicative of maternal deﬁciency but reﬂect entrenched structural and institutional inequities that shape how Māori women experience pregnancy, maternity care, and early parenting.

Counties Manukau has one of the highest birth rates in Aotearoa, with a substantial proportion of births to Māori and Paciﬁc whānau. For many wāhine Māori in this region, pregnancy occurs within social and economic contexts shaped by housing instability, ﬁnancial strain, and intergenerational trauma linked to colonisation and systemic disadvantage (3, 4). These realities intersect with maternity and child protection systems that disproportionately monitor Māori mothers (5) generating fear, guarded disclosure, and delayed engagement with care.

Pregnancy was reframed as whakapapa continuity rather than risk management.

“They talked about my baby like she already mattered. Not like a ﬁle.”

Professional participants observed strengthened mana within whānau navigating statutory systems.

Within this context, Māori mothers are frequently positioned as subjects of risk rather than as holders of knowledge, strength, and relational authority.

**Figure 1:**
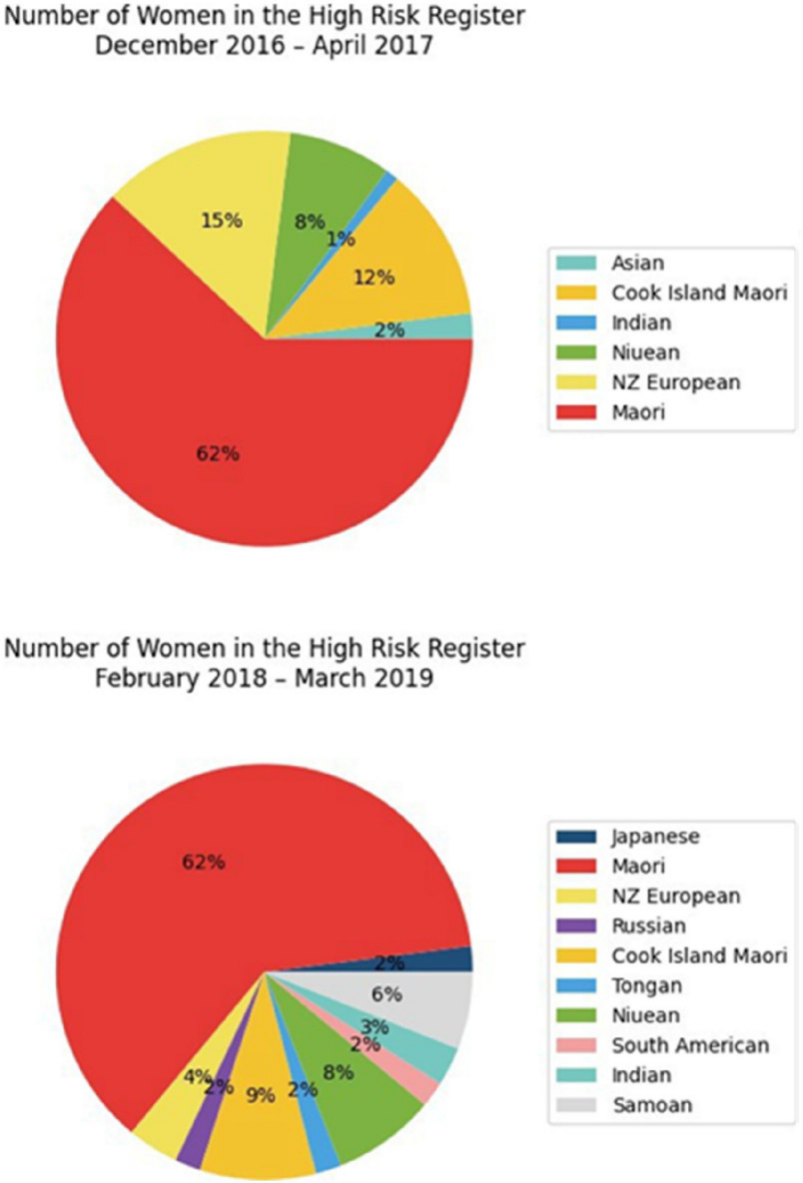
Ethnicity of women identiﬁed on the Counties Manukau high-risk register at the time of data collection. Data are derived from the author's original service-level dataset. At the time the data were collected, Māori comprised approximately.

Surveillance mechanisms such as Social Work Alerts and “high-risk” classiﬁcations are often applied without meaningful reassessment of current circumstances, reinforcing deﬁcit assumptions and limiting opportunities for trust-based engagement. From 2016 to 2019 Māori women made up between 62% and 64% of antenatal mothers assessed at high-risk Figure 1.

**Figure 2:**
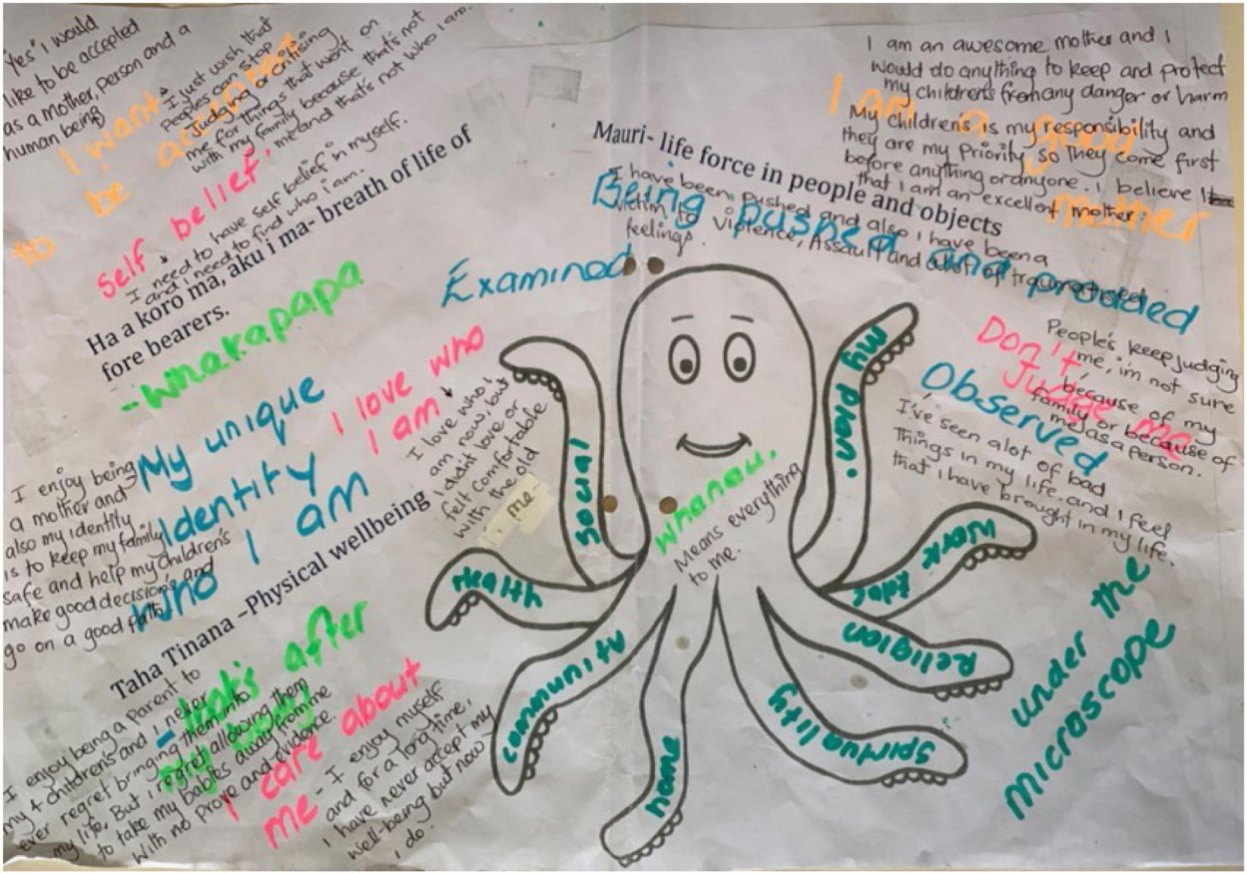
Participant-created Te Wheke wellbeing reflection illustrating interconnected dimensions of identity, whānau, and wellbeing.

**18% of the total Counties Manukau population** (1, 19). This population denominator is provided to contextualise the observed disproportionality.

### Te Waiora: A Māori-led antenatal support model

Te Waiora HEAL is grounded in te ao Māori, understanding wellbeing as interconnected across wairua (spiritual), hinengaro (emotional), tinana (physical), and whānau dimensions (6, 7).

Unlike mainstream biomedical pathways structured around risk stratiﬁcation, Te Waiora operates through:

- Sustained relational continuity- our workday was adaptable depending on changes such as environmental, legal and health needs. “we did what we said we will do,that was important for the mother”. (Kaimahi,2020). A woman was arrested and placed in the cells; we transported the baby to the cells after work hours to ensure the mother could breast feed her baby.
- Home-based and community engagement-we worked across multiple agencies often associated with surveillance, Kaimahi may attend a midwife appointment and Child protection meeting on the same day.
- Whānau-inclusive planning- whānau were included as part of the care and support we provided and at the request of the mother.
- Transparent discussion of statutory processes. We always made the mother aware that we would advise them of any contact a services was making with us and the purpose (unless legally [prohibited) in which case we would immediately move to support the mother.
- Strength-based documentation. All documentation was considered strength based to evidence the supports the mother was receiving and ensuring services had up to date information. Mothers were encouraged to use any form of communication to relay what they wanted to say, including art, written or verbal communication and they were always assured their thoughts would be treated with dignity.

The purpose of this study was to explore how wāhine Māori and associated practitioners experienced kaupapa Māori antenatal care delivered through Te Waiora, and to contribute qualitative evidence regarding Indigenous-led approaches to maternal wellbeing.

### Indigenous-led maternity care in international contexts

International literature consistently demonstrates that Indigenous-led, community-governed maternity models are associated with improved engagement, enhanced perceptions of safety, and strengthened relational continuity for Indigenous mothers. Studies from Australia, Canada, and the United States highlight the importance of culturally grounded care, Indigenous workforce leadership, and whānau-centred approaches in contexts where Indigenous women experience ongoing surveillance through health and child protection systems (8–10, 16).

The World Health Organization has similarly recognised respectful, culturally safe maternity care as critical to addressing persistent maternal health inequities globally (11, 12). These ﬁndings align with critiques of biomedical, risk-focused maternity models that prioritise surveillance over relationship and often contribute to disengagement and retraumatisation (13).

Despite this growing international evidence base, there remains limited published research detailing kaupapa Māori antenatal practice grounded in lived experience and service-based delivery in Aotearoa New Zealand. This study contributes to that gap by presenting Māori mothers’ narratives of engagement with Te Waiora, situated within both local kaupapa Māori praxis and broader Indigenous maternal health scholarship.

### Theoretical foundation: kaupapa Māori and the HEAL model

Kaupapa Māori theory asserts that Māori worldviews, values, and relational ethics provide legitimate and complete foundations for both practice and inquiry (6, 14). Within te āo Māori, wellbeing is understood as collective and relational, embedded within whakapapa, whanaungatanga, and manaakitanga.

The HEAL framework—Honesty, Empowerment, Aspirations, and Learning—was co-developed by Dianne Te Tau and Matua Jon Royal as a relational practice model grounded in kaupapa Māori. HEAL does not assess, rank, or score mothers.

Instead, it prioritises presence, transparency, and relational continuity, with safety understood as something that emerges through trust rather than documentation or compliance. To use this model a training package has been developed and is accessible through the Te Waiora website.

### Te Waiora: A Māori-led antenatal support model

Te Waiora HEAL is a Māori-led antenatal support model grounded in te ao Māori. Wellbeing is understood as collective and interconnected across wairua (spiritual), hinengaro (emotional), tinana (physical), and whānau dimensions (6, 7).

Risk is not dismissed. Rather, it is contextualised within relational knowledge, current supports, and demonstrated change. The orientation shifts from institutional liability management to mana-enhancing partnership.

A antenatal Mother found using the HEAL model allowed her to express her true self (2018).

1. Te whānau – the family
2. Waiora – total wellbeing for the individual and family
3. Wairuatanga – spirituality
4. Hinengaro – the mind
5. Taha tinana – physical wellbeing
6. Whanaungatanga – extended family
7. Mauri – life force in people and objects
8. Mana ake – unique identity of individuals and family
9. Hā a koro ma, a kui ma – breath of life from forbearers
10. Whatumanawa – the open and healthy expression of emotion.

Dr Rose Pere (7) conceptualises Te Wheke using the octopus as a metaphor for interconnected dimensions of wellbeing, including wairuatanga, hinengaro, whanaungatanga, mana ake, mauri, and others (see Figure 2).

### The HEAL framework in practice

HEAL—Honesty, Empowerment, Aspirations, Learning—guides daily practice.

#### Honesty

Kaimahi openly discuss statutory pathways, potential alerts, and system processes.

Transparency replaces guarded professional language.

#### Empowerment

Wāhine are supported to attend meetings prepared, speak in court if required, and co-design antenatal plans.

#### Aspirations

Conversations centre on parenting goals, whakapapa, and long-term vision rather than compliance metrics.

#### Learning

Practical support includes navigating housing systems, budgeting assistance, and understanding maternity processes. Kaimahi also engage in reﬂective supervision grounded in kaupapa Māori ethics.

HEAL is not a checklist. It is a relational ethic enacted consistently over time.

“You didn't speak for me. You helped me speak for myself.” (mother 2018)

## Methodology study design

This study employed a Kaupapa Māori qualitative research design, grounded in the understanding that Māori are the experts of their own experiences and that knowledge is generated through relationship, respect, and shared humanity (14).

### Participants and sampling

Purposive sampling was used to include individuals directly involved in Te Waiora antenatal support. Participants comprised:

- Seven wāhine Māori who engaged with Te Waiora during pregnancy or the early post-natal period (one participant exited prior to completing data collection due to whānau matters)
- Two midwives practicing in the Counties Manukau region
- Two community health workers
- One senior social work advocate
- Two legal advocates involved in family court pathways

Participants were recruited through existing professional and community relationships. All participation was voluntary. It is acknowledged that participants may disproportionately represent those who experienced positive engagement with the model; this is addressed as a study limitation.

### Data collection

Semi-structured interviews were conducted between 2019 and 2021 in locations chosen by participants, including homes, community clinics, and health service settings. Interviews ranged from 45 min to two hours and were conversational and relational in style, consistent with kaupapa Māori practice.

### Ethics and consent

Ethical approval was obtained through the New Zealand Health and Disability Ethics Committee in November 2019, with locality and cultural approval through the Counties Manukau Health Ko Awatea Research Ethics process. Written informed consent was obtained in all cases, with participants retaining the right to withdraw at any time.

### Data analysis

Data were analysed using a general inductive approach informed by Charmaz (15) and kaupapa Māori interpretive principles (4). Analysis prioritised context, relational dynamics, and meaning making rather than behavioural categorisation. Reﬂexive journaling and peer review supported analytic rigor.

Findings are presented as experiential accounts rather than claims of causal effectiveness.

### Limitations

The study was conducted by researchers and practitioners known to participants, consistent with kaupapa Māori principles of trust and whanaungatanga. While this relational proximity supported emotional safety, it may also have inﬂuenced how participants described their experiences.

Findings should therefore be interpreted as situated, relational accounts rather than neutral or detached observations.

### Findings

Participants described a trajectory from fear and guarded engagement toward restored trust and conﬁdence. Surveillance-based maternity practices were experienced as silencing and destabilising, while Māori-led, relational care was described as restoring mana, agency, and connection.

“It didn't matter who it was, they’d pick me up, take me to appointments, check on me. That support helped me so much.” (Woman receiving care 2019)

Participants consistently emphasised the importance of sustained presence, honesty, and cultural continuity in shaping their willingness to engage, disclose challenges, and remain connected with their pēpi and whānau. These narratives illustrate lived experience and meaning-making rather than system-level outcomes.

Professional participants similarly described Te Waiora as supporting relational engagement and reducing fear within antenatal and child protection contexts.

“The tautoko (support) provided allowed this whānau to maintain and build their mana in a confusing and confronting system.” (Legal advocate)

## Discussion

This study contributes qualitative insight into how wāhine Māori experience Indigenous led, kaupapa Māori antenatal care within a maternity and child protection landscape shaped by surveillance, risk classiﬁcation, and historical mistrust. The ﬁndings demonstrate that participants experienced Te Waiora as relationally safer, more consistent, and more supportive than mainstream maternity pathways characterised by monitoring and deﬁcit assumptions. Importantly, these ﬁndings reﬂect participant interpretations of safety, trust, and engagement rather than object

Linked assumptions were often privileged over present-day context, relational change, or strengthened supports. This echoes broader critiques of risk-based child protection and maternity systems, which demonstrate that predictive models frequently reproduce racialised inequities while failing to account for relational and structural determinants of wellbeing (5, 13).

“They told me exactly what would happen. No surprises. That made me feel strong enough to go.”

It is important to acknowledge that surveillance-oriented maternity practices persist, in part, because they are embedded within institutional logics of liability, standardisation, and risk aversion. From a system perspective, alerts, ﬂags, and documentation provide a sense of procedural safety and defensibility.

However, this study suggests that such approaches may produce relational unsafety for Māori mothers, undermining the very outcomes they seek to protect. Understanding this tension is critical: the issue is not individual practitioner intent, but the cumulative impact of systems designed to prioritise institutional risk management over relational trust.

“Before, I felt like they were waiting for me to mess up. With Te Waiora, I felt like they were waiting for me to succeed.”

“The decision to allow the baby to leave the hospital with the mother was made because the safety of the child and mother was demonstrated through the mother’s commitment to make the necessary changes and the ongoing support was clear.” *(Oranga Tamariki Supervisor, 2019)*

This study does not claim that Te Waiora produces measurable reductions in statutory intervention or deﬁnitive improvements in maternal or neonatal outcomes. Rather, it offers qualitative evidence that wāhine Māori experienced kaupapa Māori antenatal care as fundamentally different in how safety, support, and authority were enacted. The absence of disconﬁrming cases limits transferability, and participants may disproportionately represent those who experienced positive engagement.

scholarship by illustrating how kaupapa Māori practice operates in everyday antenatal contexts. Kaupapa Māori is often articulated at a philosophical level; this study demonstrates how its principles are operationalised through relational continuity, cultural accountability, and the deliberate refusal of deﬁcit framing. In this sense, Te Waiora is not positioned as an “alternative” to mainstream care, but as an example of how Indigenous knowledge systems enact care differently when given space to lead.

Finally, these ﬁndings have implications for policy and practice. If maternity and child protection systems are serious about improving equity, attention must shift from solely reﬁning risk tools toward examining how institutional practices themselves generate fear, disengagement, and inequity. Indigenous-led models such as Te Waiora highlight the importance of resourcing relational, culturally grounded care as a primary pathway rather than a supplementary service for “complex” cases. Further research incorporating broader datasets, mixed methods, and comparative designs would strengthen understanding of how such models interact with statutory systems over time.

## Conclusion

This study affirms that Māori-led, relationship-based antenatal care is experienced by wāhine Māori as restoring safety, dignity, and mana.

While further research incorporating broader datasets is required, these ﬁndings highlight the importance of Indigenous-led models in supporting maternal wellbeing and whānau integrity.

## Data Availability

The datasets presented in this article are not publicly available due to confidentiality obligations and the culturally sensitive nature of participant data. Requests for access may be considered by the author subject to ethical approval and tikanga-based governance. Requests should be directed to dianne.tetau@tewaiora.co.nz.
